# The diversity of the physics of earthquakes

**Published:** 2004-07-01

**Authors:** Hiroo Kanamori

**Affiliations:** Seismological Laboratory, California Institute of Technology, Pasadena CA 91125, USA

**Keywords:** Quantification of earthquakes, energy budget of earthquakes, radiation efficiency, rupture speed, earthquake rupture pattern, earthquake early warning

## Abstract

Earthquakes exhibit diverse characteristics. Most shallow earthquakes are “brittle” in the sense that they excite seismic waves efficiently. However, some earthquakes are slow, as characterized by tsunami earthquakes and even slower events without any obvious seismic radiation. Also, some earthquakes, like the 1994 Bolivian deep earthquake, involved a large amount of fracture and thermal energy and may be more appropriately called a thermal event, rather than an earthquake. Some earthquakes are caused by processes other than faulting, such as landslides. This diversity can be best understood in terms of the difference in the partition of the released potential energy to radiated, fracture, and thermal energies during an earthquake. This approach requires detailed studies on quantification of earthquakes and estimation of various kinds of energies involved in earthquake processes. This paper reviews the progress in this field from historical and personal points of view and discusses its implications for earthquake damage mitigation.

## 1. Introduction

Most earthquakes are caused by a failure in Earth’s interior. As the stress increases and exceeds the local strength of the material in Earth, a sudden failure occurs and elastic waves are radiated as seismic waves. This description is generally correct, but now seismologists know that the way an earthquake occurs is very diverse. Most earthquakes are like a “brittle” failure generating strong seismic waves, while some earthquakes involve slow slip motion. Also, there is evidence that some earthquakes involve highly dissipative processes with heat generation. Some earthquakes are not even caused by faulting, but caused by a large landslide. Although such diversity had been noticed qualitatively in the early days of seismology, it became clearer when the energy budget of earthquakes was studied in great detail. The energies involved in earthquakes are the potential energy *W* (elastic strain energy plus gravitational energy), the radiated energy *E_R_*, fracture energy *E_G_* (energy mechanically dissipated during an earthquake rupture), and the thermal energy *E_H_* (energy dissipated as heat). During an earthquake, the potential energy is released by Δ*W* which goes to *E_R_*, *E_G_*, and *E_H_* (i.e., Δ*W* = *E_R_* + *E_G_* + *E_H_*). How Δ*W* is partitioned into *E_R_*, *E_G_*, and *E_H_* determines the characteristics of an earthquake.

This paper is a review of the progress in this field, primarily from historical and personal points of view.

## 2. Quantification of earthquakes

The first task toward a good understanding of the physics of earthquakes is to determine the “size” of an earthquake. An earthquake is a complex physical process and no single parameter can completely represent the “size” of an earthquake.

### Magnitude scale

Since Richter (1935), many attempts have been made to introduce a parameter to define the “size” of an earthquake. In Richter (1935), the observed amplitude of seismic waves was used after corrections for the amplitude attenuation with distance have been made. Because of the limitations of the frequency response of the seismographs in old days, relatively short-period waves of a few Hz to 0.1 Hz (10 sec) were used. Richter (1935) introduced the local magnitude scale, *M_L_*, for southern California, using a relationship in the form *M_L_* = log*A* + *f*(Δ), where *A* is the maximum amplitude of seismic waves observed on the standard Wood-Anderson seismograph at a distance Δ from the epicenter. The function *f*(Δ) is determined empirically so that *M_L_* = 3 if *A* is 1 mm at a distance of 100 km. Although this is a purely empirical parameter and cannot be directly related to any specific physical parameter of the earthquake, it proved to be a very useful quantification parameter and has long been used widely not only in California but also worldwide.

The scale has been extended so that it can be determined with different types of instruments and different kinds of seismic waves. Yet, most of the scales were still empirical in the sense that they were determined from the observed amplitude and it was difficult to relate them to the physical parameters of the earthquake. Gutenberg and Richter (1942, 1955), Båth (1966) and many others attempted to relate the magnitude to more meaningful seismic source parameters such as the radiated energy, *E_R_*. The relation obtained by Gutenberg (1956),

[1] $$\text{log }E_{R}=1.5\;M_{s}+4.8\;\;\;(E_{R}\;\text{in Joule})$$

where *M_S_* is the surface-wave magnitude (the magnitude scale computed from the amplitude of seismic surface waves at a period of about 20 sec) has been used for a long time as a useful relationship, which converts the empirical parameter *M_S_* to a physical parameter *E_R_*. This practice is meaningful only if the 20 sec wave represents the overall energy spectrum. It turned out that the 20 sec wave represents the energy spectrum reasonably well for earthquakes up to *M_S_* = 7.5. For events larger than *M_S_* = 7.5, the peak of the energy spectrum is at a period much longer than 20 sec. The 20 sec wave used for *M_S_* determination can no longer represent the total energy of the radiated seismic wave; as a result, the *M_S_* scale saturates beyond *M_S_* = 8. For earthquakes with very large fault areas, for which the total amount of radiated energy is suspected to be very large, *M_S_* remains at about 8. To rectify this problem, it was important to observe seismic waves over a sufficiently broad frequency band, especially at long periods. Unfortunately, with most of the instruments available up until the mid 1950’s, it was not possible to record seismic waves accurately at periods longer than about 100 sec.

Brune and King (1967) and Brune and Engen (1969) attempted to rectify the saturation problem by determining the magnitude at about 100 sec. This partially removed the saturation problem, but it was still not clear exactly how to relate the magnitude to the radiated energy, *E_R_*.

### Seismic moment

In the early 1960’s, with the applications of elastic dislocation theory (Steketee, 1958; Burridge and Knopoff, 1964; Maruyama, 1964) and with the deployment of the Worldwide Standardized Seismic Network (WWSSN), it became possible to measure the “size” of earthquakes more quantitatively. Aki (1966) introduced the seismic moment, *M*_0_, into seismology. With the use of elastic dislocation theory, a small (compared to the wavelength of the seismic waves used) fault with the area, *S*, and displacement offset, *D*, can be represented by a force double couple with the moment of each force couple,

[2] $$M_{0}=\mu DS$$

where *μ* is the rigidity of the medium surrounding the source. The amplitude of long period (i.e., long wavelength) waves excited from this source is proportional to *M*_0_. Thus, we can determine *M*_0_ from the measurements of long-period waves after correcting for the source geometry ( i.e., type of faulting) and the propagation effects. The seismic moment is in principle a static parameter which can be used for quantification of earthquakes at very long period. It was later extended to seismic moment tensor, which can represent not only the static size of an earthquake but also the source geometry (Gilbert, 1970).

Since long-period waves can travel in the Earth without being strongly affected by attenuation and scattering during propagation, *M*_0_ can be determined most accurately among all the seismological parameters. Because of this, the determination of *M*_0_ became a standard practice in seismology.

### Seismic moment and energy

The importance of estimating the radiated energy, *E_R_*, was recognized even in the early days of seismology. For example, Galitzin (1915) tried to estimate the radiated energy from the 1911 Pamir earthquake in an attempt to understand its relation to a large landslide, which occurred at approximately the same time. Jeffreys (1923) re-examined Galitzin’s analysis and came up with an estimate which was approximately 280 times larger than that of Galitzin’s. This discrepancy illustrates the difficulty of accurately estimating the radiated energy from an earthquake.

In principle, estimation of radiated energy is straightforward. We estimate the energy flux carried by seismic waves and by integrating the flux in time and space we should be able to estimate the total radiated energy, i.e,

[3] $$E_{R}=\rho c\int_{S_{0}}\int_{-\infty }^{+\infty }{\dot{u}}^{2}(t)dtdS_{0}$$

where *S*_0_ is a spherical surface at a large distance from the source, *u*.(*t*) is the particle motion of the wave on *S*_0_ as a function of time *t*, *c* is the wave speed, and *ρ* is the density of the medium. Unfortunately, it is extremely difficult to do this in practice because we need to observe seismic waves over a wide frequency band and we need to correct for the complex effects of scattering and attenuation of waves in Earth’s interior. In those days of seismology, when the quality of seismic instruments and computational facility were limited, the large discrepancy between the estimates by Galitzin and Jeffreys was inevitable. In fact, it was still difficult in the 1970’s and is difficult even today.

An alternative method was to estimate the radiated energy from the seismic moment. Although the unit of *M*_0_ is ‘force × length’ which is ‘energy’, *M*_0_ is the strength of the equivalent force couple and is not directly related to the radiated energy, *E_R_*. *E_R_* and *M*_0_ are physically distinct parameters; *M*_0_ is a static parameter and *E_R_* is a dynamic parameter which depends on the dynamics of an earthquake. It is not possible to relate them unless we introduce some scaling relation which links the static to dynamic behaviors of an earthquake. Kanamori (1977) attempted this by using two simple assumptions: (1) stress changes associated with large earthquakes are approximately constant and (2) the stress release mechanism during an earthquake is simple where the final stress on the fault plane after an earthquake is about the same as the kinetic frictional stress during faulting. Regarding (1), Aki (1972), Abe(1975), and Kanamori and Anderson (1975) had shown that the average stress drop, Δ*σ_S_*, for large earthquakes is approximately constant at about 3 MPa (30 bars) with a range from 1 to 10 MPa. Regarding (2), Orowan (1960) discussed various stress release processes associated with earthquakes and suggested that the stress release process (2) is plausible. With these two assumptions we can show,

[4] $$E_{R}=(\Delta \sigma_{s}/2\mu )M_{0}$$

With Δ*σ_S_* = 3 MPa and *μ* = 30 GPa, a representative value in the shallow crust of Earth, this relation yields,

[5] $$C_{R}\equiv (\Delta \sigma_{S}/2\mu )=E_{R}/M_{0}=5\times 10^{-5}$$

which was used to estimate *E_R_* from *M*_0_. It turned out that *E_R_*, estimated from *M*_0_, agreed fairly well with those directly estimated earlier by Gutenberg (1956). Gutenberg (1956) had estimated radiated energies from seismic radiation for selected earthquakes to calibrate equation [1]. These earthquakes are with *M_S_* < 7.5 and the energy estimates made with relatively short-period waves (e.g., 20 sec) were believed to be fairly accurate (see also Richter, 1958).

Since the determination of *M*_0_ was accurate for very large earthquakes, the use of relation [4] for large to great earthquakes provides good estimates of *E_R_* if the two assumptions and the values of Δ*σ_S_* used above are reasonable. In a way, this has not been completely proved yet because no really great earthquake has occurred since 1966 for which this argument can be tested.

Although *E_R_* is one of the most meaningful physical parameters for quantifying earthquakes, it was not suitable for public information purposes because the public was used to a magnitude scale; it would be much easier for them to appreciate the size of an earthquake if it is given by a magnitude scale. Thus, in Kanamori’s (1977) paper, the energy was converted to the magnitude scale using [1] and the derived magnitude scale is called *M_W_*. Combining [1] and [4], the relation between *M_W_* and *M*_0_ can be written as,

[6] $$M_{W}=({\text{log}}_{10}\;M_{0})/1.5-6.07\;(M_{0}\;\text{in N-m})$$

and the *M_W_* scale is now widely used in seismology. Since it is defined by a quantity that does not saturate as the “size” increases, it is a more useful parameter than *M_S_* for quantification purposes. Note that in the old system the magnitude was determined first and then it was converted to energy using the empirical relation [1], while in the *M_W_* system the energy is estimated first and then it is expressed by a magnitude scale. Also, although the *M_W_* scale is actually computed from the seismic moment *M*_0_, with the assumptions stated above, it is based upon the energy concept. That is to say, the *M_W_* scale is an energy-based magnitude in concept, though it is often called the moment magnitude. For example, the differences between *M_W_* and *M_S_* for several great earthquakes are (the first number = *M_W_*, the second number = *M_S_*, the third number = the energy ratio): the 1960 Chilean earthquake (9.5, 8.3, 63), the 1964 Alaskan earthquake (9.2, 8.4, 16), the 1952 Kamchatka earthquake (9.0, 8.2, 16).

**Fig. 1:**
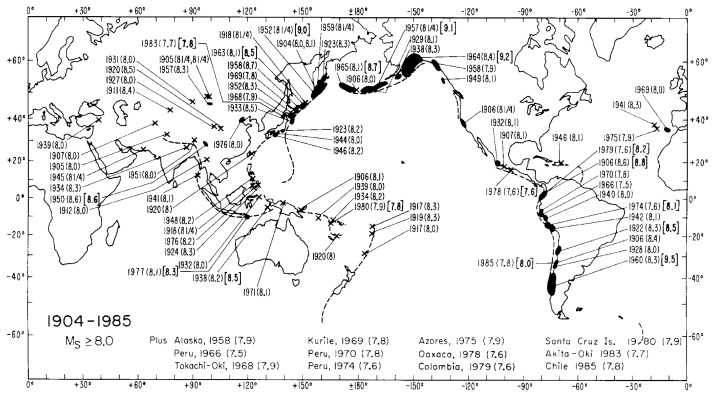
Large and great earthquakes for the period from 1904 to 1985. The surface-wave magnitude *M_S_* is given in parentheses and *M_W_* is given in brackets for the ten largest earthquakes. Dark zones indicate the rupture zones of major earthquakes. Note the difference in the level of seismicity defined by *M_W_* for different subduction zones. Although this figure shows the activity up to 1985, no earthquake with *M_W_* ≥ 8.5 has occurred since 1985.

In any case, the use of the *M_W_* scale removed the saturation problem and provided a solid framework for understanding some important features of global seismicity. Figure 1, which shows the distribution of large earthquakes along subduction zones exhibits a distinct pattern. Great earthquakes occur in South America, Alaska, the Aleutians, and Kamchatka. In contrast, earthquakes along the Marianas are much smaller. The seismicity in other subduction zones is intermediate between these groups. Although this regional variation is now generally accepted, it was not until the energy-based quantification method with *M_W_* was developed. This variation reflects the difference in the degree of inter-plate coupling at different subduction zones and has important implications for understanding the tectonic framework of global seismicity. For more details, see e.g., Kanamori (1971), Uyeda and Kanamori (1979), Ruff and Kanamori (1980), Kanamori (1986), and Scholz and Campos (1995).

**Fig. 2:**
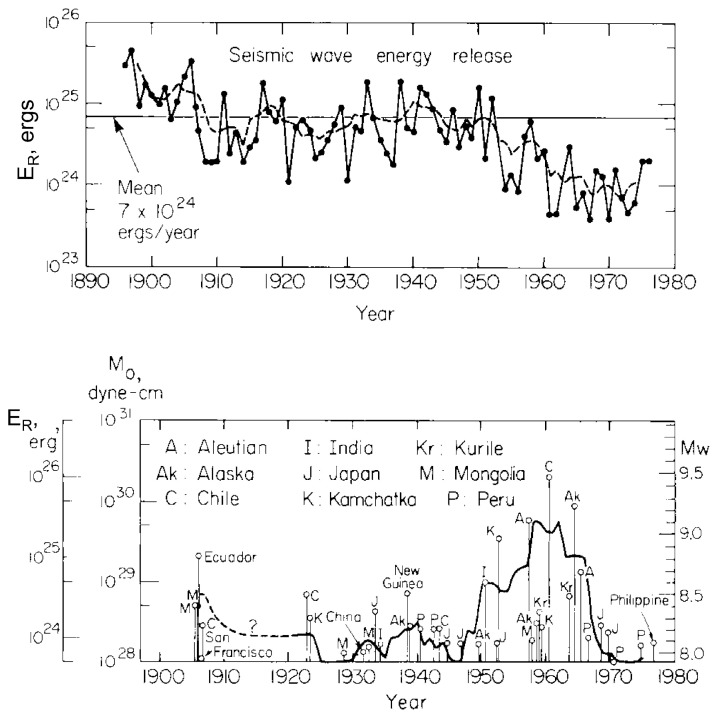
Top: Seismic wave energy radiated by earthquakes computed from *M_S_*, using equation [1]. Dashed curve shows the unlagged 5-year running average. Bottom: Radiated energy estimated from *M*_0_, using equation [4]. Although this figure shows the activity up to 1980, no earthquake with *M_W_* ≥ 8.5 has occurred since 1980. Note the drastic difference between the two, which is caused by saturation of *M_S_* for great earthquakes.

Figure 2 shows the temporal variation of earthquake energy release in the 20th century. Figure 2a and 2b show, respectively, the energy release computed with the old *M_S_* system (equation [1]) and the new *M_W_* system. Note the drastic difference which demonstrates that without the energy-based concept our understanding of global seismicity is seriously hampered.

Since the data used in Kanamori (1977) were old, the results for the individual events are subject to considerable uncertainty. Okal (1992) reassessed the seismic moments of large historical earthquakes which can be used to check the results of Kanamori (1977).

## 3. Radiated energy

As discussed above, very long-period waves (100 sec or longer) could be used to estimate the radiated energy *E_R_* using equation [4], but the method is still indirect and involves large uncertainties. Also, the scaling relation used in equation [4] varies from event to event so that the constant *C_R_* may depend on the events being studied. Thus, it was desirable to estimate *E_R_* directly using [3]. However, because of the limited data and computational facility, it was difficult to estimate *E_R_* accurately using [3], except for a few cases. Only in the late 1970’s, it became possible to estimate *E_R_* using high-quality seismic data obtained with modern seismograph systems like the Worldwide Standardized Seismographic Network (WWSSN) (Kikuchi and Fukao, 1988) and other broadband seismic networks such as IRIS and Geoscope. Boatwright and Choy (1986) and Choy and Boatwright (1995) developed a method to estimate *E_R_* from seismic body waves recorded with modern digital seismographs. Many other attempts have been made using both global and regional seismic data (e.g., Kanamori *et al*., 1993b; Singh and Ordaz, 1994; Pérez-Campos *et al*., 2003; Venkataraman and Kanamori, 2004).

However, even with the most modern data and methods, the uncertainty of the estimates of *E_R_* is still about a factor of 2 to 3. Nevertheless, compared with the situation decades ago, now the quality of *E_R_* estimates is becoming just good enough to make more quantitative discussions on the diversity of the physical mechanisms of earthquakes on the basis of energy budget, as will be discussed in the next section.

## 4. Fracture energy

Fracture energy is the energy that is dissipated mechanically during seismic faulting. Several methods can be used to estimate *E_G_*.

### Seismological method 1 — Energy method

If we model an earthquake process as a stress release process in which the stress on the fault plane drops from the initial stress *σ*_0_ to the final stress *σ*_1_, the total potential energy drop is given by,

[7] $$\Delta W=\frac{1}{2}\bar{(\sigma_{0}+\sigma_{1})DS}$$

where *S* is the fault area and *D* is the total offset of the fault. In the above the symbol “—” means the spatial average (Knopoff, 1958; Kostrov 1974; Dahlen 1977). This equation can be rewritten as,

[8] $$\Delta W=\frac{1}{2}\bar{(\sigma_{0}+\sigma_{1})D}S+\bar{\sigma_{1}D}S=\frac{1}{2}\bar{\Delta \sigma_{\text{S}}D}S+\bar{\sigma_{1}D}S=\Delta W_{0}+\bar{\sigma_{1}D}S$$

where,

[9] $$\Delta W_{0}=\frac{1}{2}\bar{\Delta \sigma_{S}D}S$$

Two difficulties are encountered. First, with seismological measurements alone, the absolute value of the stresses, *σ*_0_ and *σ*_1_, cannot be determined. Only the difference, or the static stress drop, Δ*σ_S_* = *σ*_0_ – *σ*_1_, can be determined. We cannot compute Δ*W* from seismic data. Second, with the limited resolution of seismological methods, the details of spatial variation of stress and displacement cannot be determined. Thus, we commonly use, instead of [9],

[10] $$\Delta W_{0}=\frac{1}{2}\bar{\Delta \sigma_{S}}\bar{D}S$$

Unfortunately, it is not possible to accurately assess the errors associated with the approximation of equation [10]. It is a common practice to assume that the spatial variations of *D* and Δ*σ_S_* are smooth enough to justify this approximation.

Although Δ*W* cannot be determined with seismological methods, Δ*W*_0_ can be computed from the seismologically determined parameters $\bar{\Delta \sigma_{S}}$, *D̄*, and *S*. In general $\bar{\sigma_{1}D}S>0$, unless a large scale overshoot on a fault plane occurs and Δ*W*_0_ can be used as a lower bound of Δ*W*. If the final residual stress *σ*_1_ is small, Δ*W*_0_ is a good approximation of Δ*W*.

**Fig. 3:**
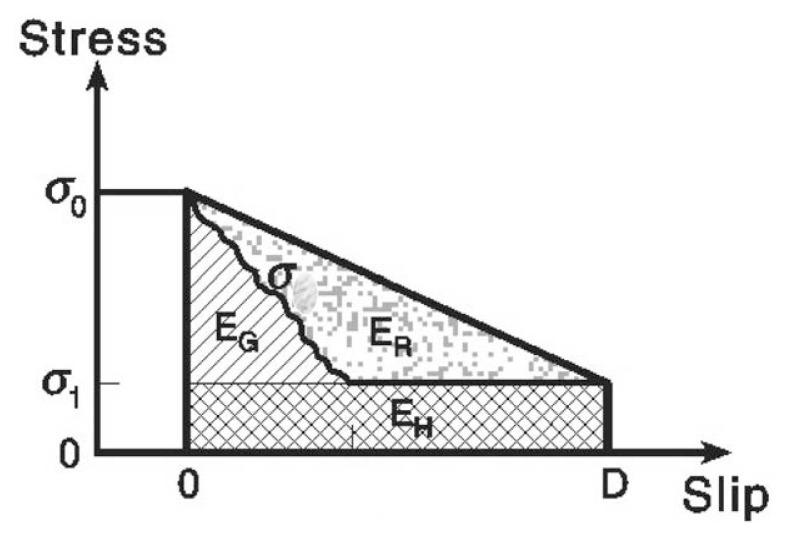
Illustration of a simple stress release model for an earthquake. Hatched, cross-hatched, and dotted areas indicate the fracture energy, thermal energy, and radiated energy, respectively. The figure is shown for unit area of the fault plane.

It is important to note that we can determine two kinds of energies, radiated energy, *E_R_*, and the lower bound of the potential energy change, Δ*W*_0_, with seismological data and methods. These two energies play an important role for understanding the physics of earthquakes. The energy partition can be most conveniently illustrated by a stress-vs-slip diagram shown in Fig. 3. In Fig. 3, the trapezoidal area with the base from 0 to *D* gives Δ*W* per unit area.

If the stress drops from *σ*_0_ to *σ*_1_ and fault slip motion occurs at a constant residual stress *σ*_1_, the energy *E_F_* = *σ*_1_*DS* can be considered as the frictional energy during faulting. In this case, this energy is dissipated on the fault surface and does not contribute to the dynamic process of faulting involving the fault tips. We often assume that the entire frictional energy defined this way goes to heat, and write *E_H_* = *E_F_*, and call it the thermal energy. from [10], we can regard Δ*W*_0_ = Δ*W* – *E_H_*, which is given by the triangular area, with the base from 0 to *D*, as the energy available for the dynamic process of faulting. The difference between Δ*W*_0_ and *E_R_* measured with the methods described earlier is the energy mechanically dissipated (not radiated) during faulting, which is the fracture energy, *E_G_* (i.e., *E_G_* = Δ*W*_0_ – *E_R_*). In the widely used slip-weakening model, *E_G_* is given by the shaded area in Fig. 3. We define the radiation efficiency *η_R_* by (Husseini, 1977),

[11] $$\eta_{R}=E_{R}/\Delta W_{0}=E_{R}/(E_{R}+E_{G})$$

which gives the ratio of the radiated energy to the energy available for mechanical process, Δ*W*_0_. This parameter, *η_R_*, is useful for characterizing the dynamic behavior of an earthquake. If *η_R_* = 1, the earthquake is very efficient in radiating energy. If *η_R_* = 0, the entire Δ*W*_0_ is dissipated mechanically and no energy is radiated. Using *M*_0_ and Δ*σ_S_*, we can write *η_R_* as,

[12] $$\eta_{R}=E_{R}/\Delta W_{0}=E_{R}/(\Delta \sigma_{S}SD/2)=\frac{2\mu }{\Delta \sigma_{S}}\frac{E_{R}}{M_{0}}$$

**Fig. 4:**
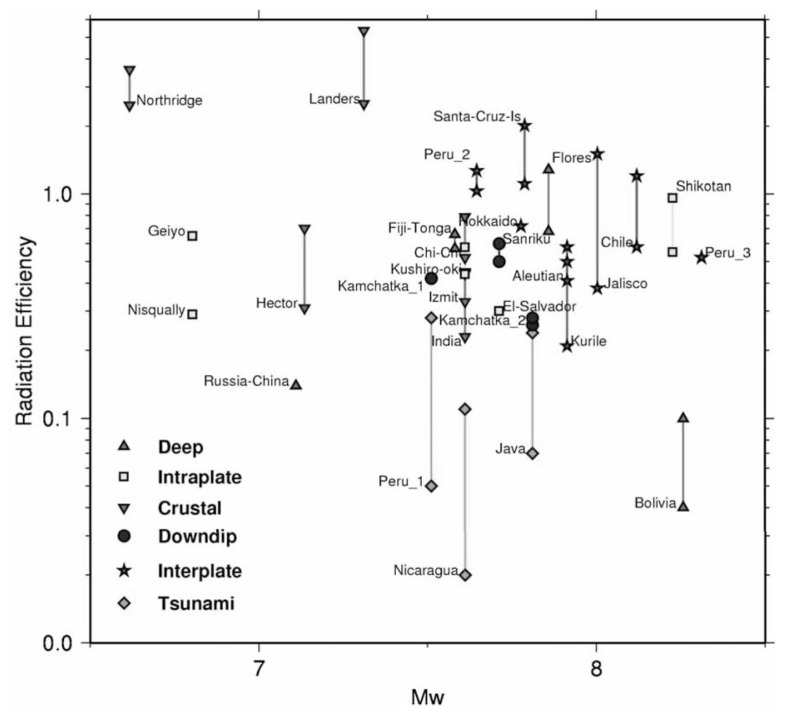
Radiation efficiency *η_R_* = *E_R_* /(*E_R_* + *E_G_*) as a function of *M_W_*. The different symbols show different types of earthquakes as described in the legend. Most earthquakes have radiation efficiencies greater than 0.25, but tsunami earthquakes (Peru_1, Java, Nicaragua) and two of the deep earthquakes (the Bolivia earthquake and the Russia-China border earthquake) have small radiation efficiencies. (From Venkataraman and Kanamori (2004)).

All the quantities on the right hand side of this equation are macroscopic, seismological parameters which can be determined from observations. Figure 4 shows the results taken from Venkataraman and Kanamori (2004). The radiation efficiency, *η_R_*, of most earthquakes is larger than 0.25. Tsunami earthquakes are earthquakes with slow deformation at the source, which generate tsunamis disproportionately large for the earthquake magnitude. They have small radiation efficiencies (< 0.25). The two deep earthquakes, the 1999 Russia-China border event and the 1994 deep Bolivia earthquake, have small radiation efficiencies, *η_R_* ≈ 0.1.

For a few earthquakes the computed *η_R_* is larger than 1. This is probably due to the errors in the estimates of radiated energy and stress drops. Even today, the estimates of these macroscopic parameters, *M*_0_, *E_R_*, and Δ*σ_S_* are still subject to considerable uncertainties; as a result, the estimate of *η_R_* is inevitably uncertain. In fact, Kikuchi’s (1992) result is significantly different from that shown in Fig. 4. This discrepancy has not been completely resolved yet.

### Seismological method 2 — Rupture speed

The radiation efficiency, *η_R_*, can also be estimated from the rupture speed, *V*, of faulting. In general a rupture in solid materials propagates at a speed which is some fraction of the shear-wave speed, *β*, in the material. In most laboratory tests, tensile fracture propagates at about 40% of the shear-wave speed or less. In contrast, the rupture speed of seismic faulting is generally much faster. In most cases, it is comparable to *β* or, in some cases, even faster. In general if *V* is very low, the rupture is almost quasi-static and no energy is radiated, i.e., *η_R_* ≈ 0. On the other hand if *V* ≈ *β*, little energy is mechanically dissipated near the extending edge of the fault, and *η_R_* ≈ 1. Theories by Mott (1948) and Kostrov (1966) (also see Rose, 1976; Freund, 1989; Eshelby, 1969; Fossum and Freund, 1975; Freund, 1972) suggest that,

[13] $$\eta_{R}\approx {\left(V/\beta \right)}^{1\;\text{to }2}$$

**Fig. 5:**
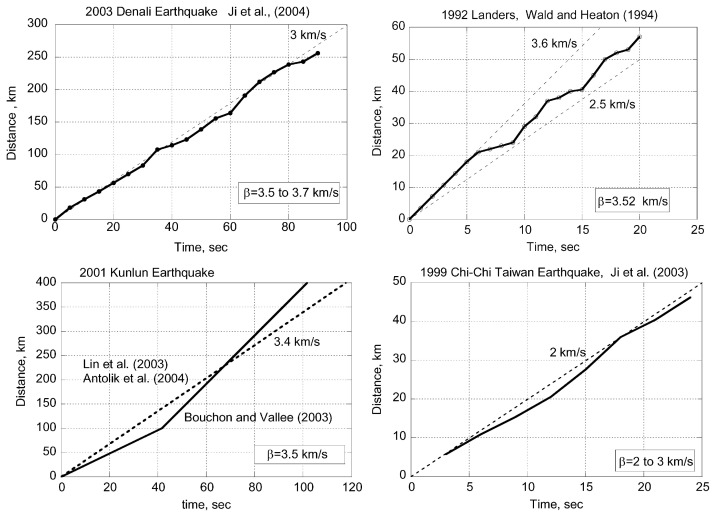
Time-distance curves for rupture propagations for recent earthquakes. These figures were constructed from the figures of rupture patterns published in the following references: Denali earthquake (Ji *et al*., written communication, 2004), Kunlun earthquake (Bouchon and Vallée, 2003; Lin *et al*., 2003, Antolik *et al*., 2004), Chi-Chi earthquake ( Ji *et al*., 2003), and Landers earthquake (Wald and Heaton, 1994).

Although this should be regarded as a very approximate relation, it provides a useful means for estimating *η_R_* from the observation of rupture speed, *V*. For most large earthquakes (*V*/*β*) > 0.5 (e.g., Venkataraman and Kanamori, 2004), suggesting that *η_R_* is larger than 0.25, which is consistent with the results obtained from the energy budget discussed above. For several recent large earthquakes, detailed determinations of the rupture front have been made. Figure 5 shows the result for the 2002 Denali, Alaska earthquake, the 2001 Kunlun, China earthquake, the 1999 Chi-Chi, Taiwan earthquake, and the 1992 Landers earthquake. Because the shear-wave speed, *β*, in the crust varies as a function of depth, the rupture speed, *V*, is compared with *β* at the depth where the largest slip occurred. The value of *β* chosen is indicated in the figure. Also, note that *V* reaches the high limiting speed almost instantly, at least for the Denali, the Chi-Chi, and the Landers earthquakes. For the Kunlun earthquake, *V* in the beginning is not resolved well. For the Landers earthquake, the rupture slowed down several times during faulting when the rupture transferred to a different fault segment.

Another important observation concerning earthquake rupture speed is that for several earthquakes supershear (i.e., *V* > *β*) rupture speeds have been reported. The examples are the 1979 Imperial Valley earthquake (Archuleta, 1984), the 1999 Izmit earthquake (Bouchon *et al*., 2001), and the 2001 Kunlun earthquake (Bouchon and Vallée, 2003). Although the details are still being debated, the observation that the rupture speed is comparable to, or faster than, *β*, at least over some segments of the fault, appears well established. Supershear rupture speeds are most likely if the fracture energy, *E_G_*, is small, i.e., *η_R_* is large.

As mentioned earlier, the estimates of *η_R_* from the energy budget are still considerably uncertain. In contrast, the quality of determination of *V* is rapidly improving recently so that the estimates of *η_R_* from *V* appears more robust. The overall consistency between the results from the energy budget and the rupture speed enhances the conclusion that the radiation efficiency for most large shallow earthquakes is relatively large.

### Fracture energy and fault gouge

**Fig. 6:**
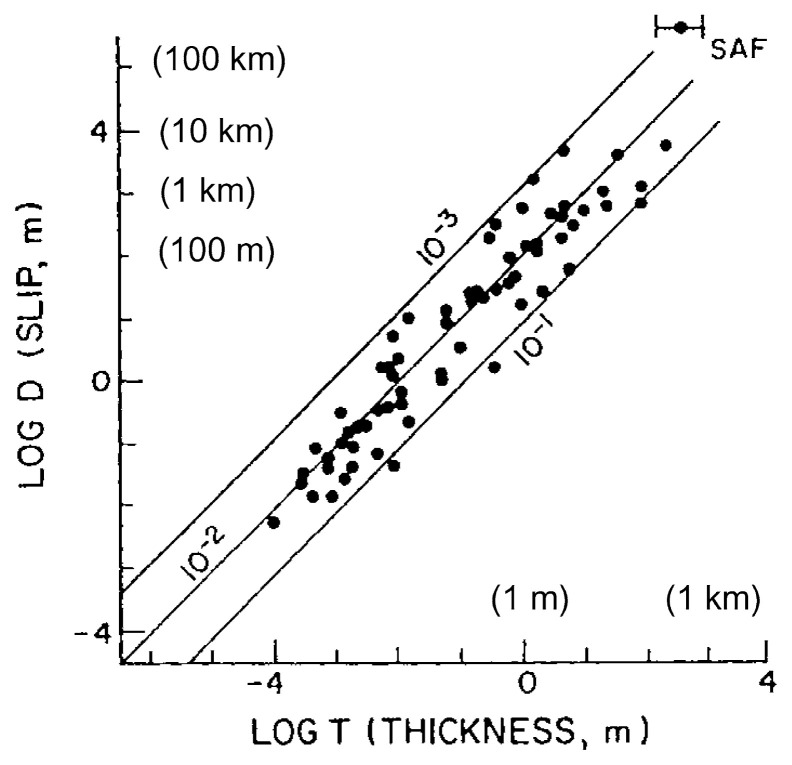
Thickness of the gouge zone, *T*, plotted versus total slip, *D*, for a collection of faults. (From Scholz, 2002)

The radiation efficiency, *η_R_*, estimated from seismological data suggests that the fracture energy, *E_G_*, for most large earthquakes is comparable to, or smaller than, the radiated energy, *E_R_*. Since the fracture energy is literally the energy used to fracture the rocks near the fault zone, it is also possible to estimate it from field data on fault-zone structures. In general, a zone of crushed rocks, called fault gouge, is observed along faults. The width of the gouge layer, *T*, has been measured by various investigators (Robertson, 1982; Otsuki, 1978). Figure 6 is taken from Scholz (2002). In Fig. 6, *D* is the total amount of fault slip over the entire life time of the fault, rather than the slip in one earthquake. In the previous sections, *D* is the slip in one earthquake. We can estimate the total fracture energy used to form the gouge layer as follows:

Suppose we consider an initially unbroken block of crustal rock. Then, after the fault slipped many times a gouge layer with a thickness *T* is formed. Let *L* be the fault length, and *H* be the width of the fault. Then *V* = *LHT* is the volume of the gouge layer. Suppose that the gouge layer consists of grains with radius *a*. The number of grains in this volume is *N* = *THL*/(4*π a*^3^/3) and the total surface area of the grains is *S_G_* = 4*π a*^2^*N* = 4*TLH*/*a* = 4*TS*/*a*, where *S* = *TH* is the fault area. If the fracture energy required to produce a new surface is *G_C_* (per unit area), then the total fracture energy associated with the formation of the gouge layer is given by,

[14] $$E_{G}=(G_{C}/2)\lambda S_{G}=2\lambda G_{C}ST/a=2\lambda G_{C}M_{0}(T/D)/\mu a$$

where *λ* is a factor to correct for the difference between the geometrical and actual shapes of the grain. Here we use *λ* = 6.6 (Wilson *et al*., 2002). If we use equation [5] as the empirical relation relating *M*_0_ to *E_R_*, we obtain,

[15] $$\eta_{R}=\frac{1}{1+\frac{2\lambda }{\mu C_{R}}\left(\frac{T}{D}\right)\left(\frac{G_{C}}{a}\right)}$$

**Fig. 7:**
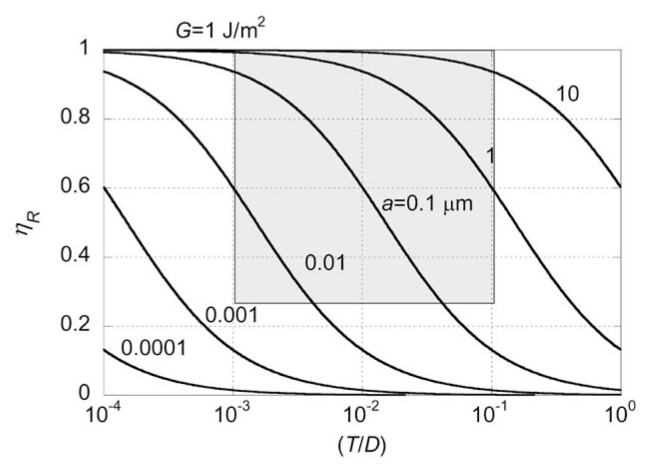
The relationship between the radiation efficiency, *η_R_*, and the gouge thickness divided by the total displacement, *T*/*D*, with the grain size of the gouge as a parameter. The shaded area shows the range of *η_R_* estimated from seismological data and of (*T*/*D*) estimated from field data. The specific fracture energy for the gouge material, *G_C_*, is assumed to be 1 J/m^2^.

The specific fracture energy, *G_C_*, for minerals and rocks ranges from 0.1 to 10 J/m^2^ (Friedman *et al*., 1972; Scholz, 2002; Lawn, 1993) and here *G_C_* =1 J/m^2^ is used as a representative value. Note that in the context of the specific slip-weakening model discussed above, the use of [5] implies *η_R_* ≈ 1, but here [5] is used just as an empirical relation relating the observed *M*_0_ to the observed *E_R_*, regardless of the model. Relation [15] is shown in Fig. 7 with the grain size *a* as a parameter.

The shaded area on Fig. 7 indicates the range of *η_R_* estimated from seismology for large shallow earthquakes and of (*T*/*D*) determined from field data (Fig. 6). If the grain size *a* is in the range from 0.01 to 10 μm, the observed field data on (*T*/*D*) and the seismologically estimated *η_R_* can be made compatible. This range covers the range of grain size of the fault gouge. Many parameters (e.g., *G_C_*) and assumptions (e.g., uniform grain size) are used in this argument, but this overall compatibility between seismological and field data supports the conclusion that *η_R_* ≥ 0.25.

Interpretation of field data involves subjective judgments, especially on the definition of fault gouge. If a new observation is made for gouge structure of a fault, Fig. 7 can be used to check the consistency between a specific model of gouge formation and seismological data.

## 5. Thermal energy

**Fig. 8:**
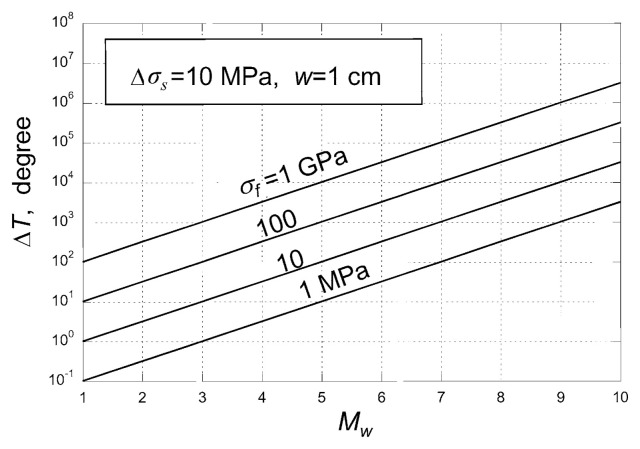
Predicted temperature rise, Δ*T*, in a fault zone as a function of magnitude, *M_W_*, with the frictional stress, *σ_f_*, as a parameter. The static stress drop, Δ*σ_S_*, is assumed to be 10 MPa (from Kanamori and Heaton, 2000).

It is not possible to estimate the magnitude of the frictional stress during faulting directly with seismological methods and we cannot estimate the thermal energy directly. Nevertheless, this problem has been discussed by Terada (1930), Jeffreys (1942), and many others. The following is a simplified discussion on a gross thermal budget during faulting under a frictional stress, *σ_f_* (= *σ*_1_). If the entire frictional energy is converted to heat, the total heat generated during faulting is *Q* = *σ_f_ DS*. Then, the average temperature rise, Δ*T*, is given by,

[16] $$\Delta T=Q/C\rho Sw=\sigma_{f}D/C\rho w$$

where *C* is the specific heat, *ρ* is the density, and *w* is the thickness of the layer along the rupture plane in which heat is distributed. In general we can relate *D* to *M_W_* (the larger the magnitude, the larger the displacement) and compute Δ*T* as a function of *M_W_*. Figure 8 shows Δ*T* for the case with *w* = 1 cm.

If *σ_f_* is about 10 MPa (this is comparable to the stress drop during earthquakes), the effect of shear heating is significant. If the thermal energy is contained within a few cm around the slip plane during a seismic slip, the temperature would easily rise by 100 to 1000 °C during a moderate-sized earthquake.

Although how thick the slip zone at depth is not well understood, Sibson (2003) suggests that *w* can be as small as a few mm to a few cm. Then, even for the modest frictional stress assumed above, the temperature in the fault zone can become very high during faulting. If the temperature is very high, it is possible that various lubrication processes can operate to reduce friction (e.g., Sibson, 1973; Lachenbruch, 1980; Brodsky and Kanamori, 2001). Fialko (2004) discusses the details of the temperature effects on dynamic crack propagation. If friction drops, then the temperature will drop and eventually fault motion may occur at some equilibrium state at relatively modest friction. Although this conclusion depends on the estimate of *w* and the assumed lubrication mechanisms, it is possible that kinetic friction during large earthquakes is relatively small and the total frictional energy, *E_H_*, is relatively small. This problem, however, is still actively debated (e.g., Scholz, 2000; McGarr, 1999).

## 6. Different types of earthquakes

As we discussed above, we can characterize earthquakes on the basis of partition of the potential energy Δ*W* to *E_R_*, *E_G_*, and *E_H_*.

### Large shallow earthquakes

As shown in Fig. 4, for most large shallow earthquakes, *η_R_* is fairly large, most likely > 0.25. This conclusion seems to be substantiated by the relatively fast rupture speed, as shown in Fig. 5. In this sense, a rupture of most shallow earthquakes is considered “brittle”. In general, “brittle” failure involves little resistance and once it starts it is hard to stop.

### Tsunami earthquakes, slow earthquakes, and silent earthquakes

Some earthquakes are not “brittle” in this sense. For example, one such class of earthquakes are “tsunami earthquakes”. Tsunami earthquakes are those earthquakes for which tsunami is disproportionately large for the seismic magnitude (Kanamori, 1972, Fukao, 1979; Pelayo and Wiens, 1992). This behavior can be explained if the deformation associated with the earthquake is slower than ordinary earthquakes. In terms of the energy partition, the radiation efficiency, *η_R_*, of tsunami earthquakes is much smaller than those of ordinary earthquakes, as shown in Fig. 4. Examples of tsunami earthquakes are the 1896 Sanriku, Japan earthquake (Tanioka and Satake, 1996) and the 1946 Unimak Is. earthquake in the Aleutians (Johnson and Satake, 1997). These earthquakes caused some of the largest tsunamis in history, but their earthquake magnitudes are about 7.5, a modest value. More recent examples are the 1992 Nicaragua earthquake (Kanamori and Kikuchi, 1993; Satake, 1994) and the 1994 Java earthquake (Tsuji *et al*., 1995). For these events, analyses of high-quality modern seismic data demonstrated that the radiated energy, *E_R_*, was indeed small compared with the available mechanical energy, Δ*W*_0_.

Slow earthquakes also occur on land. In this case, the effect is not obvious and their detection is more difficult. However, the existence of such slow and even almost silent earthquakes has been firmly confirmed with the analysis of strain meters and the Global Positioning System (GPS) (Linde *et al*. 1996; Dragert *et al*., 2001; Kostoglodov *et al*., 2003; Miyazaki *et al*., 2003). These earthquakes do not excite seismic waves and, in that sense, *η_R_* ≈ 0.

### Deep earthquakes

For the 1994 Bolivian earthquake (*M_W_* = 8.3, depth = 635 km), the largest deep earthquake ever recorded, the source parameters could be determined well enough to investigate the energy budget (Kanamori *et al*., 1998). The result showed that Δ*W*_0_ = 1.4 × 10^18^ J and *E_R_* = 5 × 10^16^ J, which is only 3 % of Δ*W*_0_, and the difference Δ*W*_0_ – *E_R_* = 1.35 × 10^18^ J, was not radiated and must have been deposited near the focal region, probably in the form of fracture energy in addition to the frictional energy. This energy 1.35 × 10^18^ J is comparable to the total thermal energy released during large volcanic eruptions, such as the 1980 Mount St. Helens eruption. In other words, fracture and thermal energy, at least comparable to that released by a large volcanic eruption, must have been released in a relatively small focal region of about 50 × 50 km^2^, within a matter of about 1 min. The elastic part of the process, *i.e*. the earthquake observed as seismic waves, is only a small part of the whole process. Therefore, the Bolivia earthquake should be more appropriately viewed as a thermal process rather than an elastic process. How much of the non-radiated energy goes to heat depends on the details of the rupture process, which is unknown. However, it is likely that a substantial part of the non-radiated energy was used to significantly raise the temperature in the focal region. The actual temperature rise, Δ*T*, also depends on the thickness of the fault zone, which is not known; if it was of the order of a few cm, the temperature could have risen to above 10,000 °C (Fig. 8). Whether other deep earthquakes are like this or not is not known. In fact, several studies suggest that although *η_R_* is small for some events, in general it is not as extreme as for the Bolivian earthquake (Tibi *et al*., 2003; Estabrook, 2004). However, the resolution of seismic methods is limited and only for the very large events, like the Bolivian earthquake, can we constrain the source parameters well. As a result, although the conclusion for the Bolivian earthquake is fairly solid, more general conclusions must await further studies.

### Other types of earthquakes

It is now generally accepted that most earthquakes are caused by faulting, a sudden shear fracture in the Earth’s crust and mantle. However, some earthquakes are caused by processes other than faulting. These earthquakes are generally called non-double couple earthquakes. One of the most spectacular events is the long-period excitation by a large landslide associated with the 1980 Mt. St. Helens eruption. During this eruption, large blocks on the north flank of the mountain slid northward over more than 11 km. The total volume involved was more than 1.5 km^3^. The large slide excited long-period seismic waves which were recorded at stations all over the world. The radiation patterns of surface waves excited by this event clearly indicated that the source is not a faulting, but a landslide (Kanamori and Given, 1982; Kanamori *et al*., 1984; Burger and Langston, 1985; Kawakatsu, 1989). The horizontal acceleration and deceleration of the landmass along the slope exerted an equivalent single force on the ground, which excited seismic waves. Although the model we introduced above for faulting cannot be used, the amount of radiated energy was obviously a very small fraction of the total energy involved. In this sense, the radiation efficiency is very small.

A landslide, or slumping, has also been suspected to be the source of some earthquakes (e.g., the 1929 Grand Banks earthquake (Hasegawa and Kanamori, 1987) and the 1998 Papua New Guinea earthquake (Synolakis *et al*., 2002; Tappin *et al*., 2001).

Another source of excitation of earthquakes comes from the atmosphere. During the large eruption of Mount Pinatubo in the Philippines in 1991, unusual harmonic oscillations with a period of about 230 sec and with a duration of a few hours were recorded by seismographs at many stations in the world (Kanamori and Mori, 1992; Widmer and Zürn, 1992). Such harmonic waves had not been recognized before and their cause was not understood immediately. The subsequent investigations (Kanamori and Mori, 1992; Kanamori *et al*., 1994) demonstrated that the atmospheric acoustic oscillations set off by the eruption excited seismic Rayleigh waves (surface waves) at points near the volcano, which were observed worldwide with seismographs.

Some earthquakes in volcanic areas often contain components which are most likely caused by some magmatic or geothermal processes, such as magma injection or fluid injection. Some notable examples are the 1984 Torishima earthquake (Kanamori *et al*., 1993a), a series of earthquakes in Long Valley, California, (Dreger *et al*., 2000), and many earthquakes associated with the 2000 Miyakejima, Japan sequence involving dike injection. The non-double couple earthquakes are discussed in detail in Julian *et al*. (1998) and Miller *et al*. (1998).

### Implications

In the context of the model presented above, slow and silent earthquakes must involve processes which are highly dissipative. The question is, what is causing dissipation. Tsunami earthquakes probably involve the deformation of a large amount of water-saturated sediment. Partly because of the large amount of sediment deformed and partly because of phase changes of fluids, which are heated by fault motion, it is likely that a large amount of energy is dissipated during rupture of tsunami earthquakes.

A recent detailed study of the relationship between silent earthquakes and the crust-mantle structure in the Nankai trough (Kodaira *et al*., 2004) suggests that a high pore pressure increases the sliding stability, which is equivalent to increasing the fracture energy, *E_G_*. In the Cascadia subduction zone, episodic slow slip correlates in time with increase in low-frequency tremors (Rogers and Dragert, 2003). Although the mechanism of either the episodic slow slip or the low-frequency tremor is not fully understood yet, it is likely that fluids are involved in both.

These observations are relevant to the question regarding why plate motion is accommodated by seismic slip in some subduction zones (e.g., southern Chile) and by aseismic slip in other subduction zones (e.g., the Marianas).

The research on these newly found processes has just begun and it would be useful to investigate these problems from the energy budget involved in the processes.

## 7. Rupture behavior of large shallow earthquakes

As Fig. 4 demonstrates, most shallow earthquakes seem to be fairly efficient in energy radiation, i.e., *η_R_* > 0.25. The kinetic friction during faulting can be low, at least after a significant amount of slip motion has taken place. However, this statement is not universally accepted and is debated in the seismological literature. For example, Scholz (2000) and McGarr (1999) argue that kinetic friction is large even for large earthquakes, implying that the absolute stress on the fault plane is much higher than earthquake stress drops. Wilson *et al*. (2004) argue that the grain size of fault gouge has been significantly underestimated in previous studies and the fracture energy associated with the San Andreas fault can be a significant part of earthquake energy budget. The problem is far from being resolved.

Here, we present some observational data for large shallow earthquakes and interpret them in terms of the conceptual model presented in this paper (i.e., large *η_R_* and low friction), with a caveat that other interpretations from the opposing views (i.e., small *η_R_* and high friction) may be equally possible.

The rupture process is complex, which results in complex spatial and temporal slip patterns on a fault plane. Although the spatial and temporal rupture patterns are important for understanding the physics in detail, here we simplify the problem by reducing the rupture patterns to moment-rate functions. We introduced the seismic moment given by *M*_0_ = *μ DS* as a static parameter for an earthquake where *D* and *S* are determined when the earthquake is over. However, we can also define it during a seismic rupture while *D* and *S* are changing in time. In this case, *M*_0_ is given as a function of time. The rate of *M*_0_(*t*) is called the moment rate and written as *Ṁ*_0_(*t*). Qualitatively, this may be viewed as how energy is radiated from the source as a function of time. The moment-rate function behaves differently depending upon whether rupture propagation is approximately one dimensional (e.g., crustal strike-slip earthquakes and narrow thrust earthquakes) or two dimensional (e.g., large subduction-zone thrust earthquakes).

**Fig. 9:**
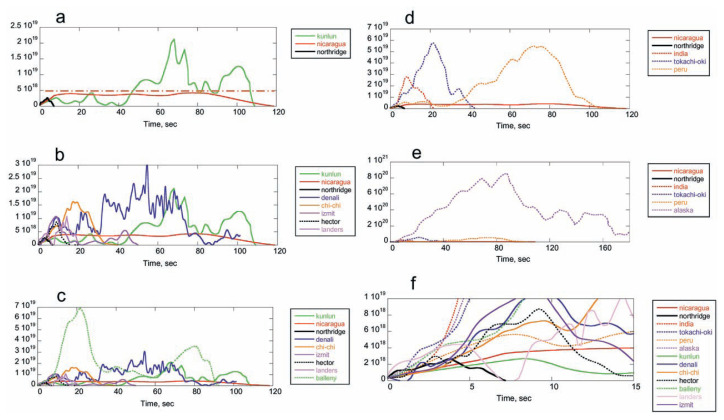
The moment rate functions *Ṁ*_0_(*t*) (unit, N–m/sec) for large shallow earthquakes. (a) The 1994 Northridge, the 2001 Kunlun, and the 1992 Nicaragua earthquakes. The dash-dot horizontal line at *Ṁ*_0_(*t*) = 5 × 10^18^ N-m/sec is shown as a reference. (b) The moment rate functions for the 2002 Denali, the 1999 Chi-Chi, the 1999 Izmit, the 1999 Hector Mine, and the 1992 Landers earthquakes are added to those in Fig. 9a. (c) The moment rate functions for the 1998 Balleny Is. earthquake is added. (d) The moment rate functions for the 2001 India, the 2003 Tokachi-Oki, and the 2001 Peru earthquakes. (e) The moment rate function for the 1964 Alaskan earthquake is added to those in Fig. 9d. (f) The first 15 s of the moment rate functions. References to the moment rate functions: 1992 Nicaragua (*M_W_* = 7.6), Kanamori and Kikuchi (1993); 1994 Northridge (*M_W_* = 6.7), Thio and Kanamori (1996); 2001 Kunlun, China (*M_W_* = 7.8), Lin *et al*., (2003); 2002 Denali, Alaska (*M_W_* = 7.9), Tsuboi *et al*. (2003) and C. Ji ( written communication, 2003); 1999 Chi-Chi (*M_W_* = 7.6), Ji *et al*., (2003); 1999 Hector Mine, California (*M_W_* = 7.1), Ji *et al*., (2002); 1992 Landers, California (*M_W_* = 7.3), Dreger (1994); 1999 Izmit, Turkey (*M_W_* = 7.6), Li *et al*., (2002); 1998 Balleny Is., Antarctica (*M_W_* = 8.1), Henry *et al*., (2000) and Hjorleifsdottir (written communication, 2003); 2001 India (*M_W_* = 7.6) and 2001 Peru (*M_W_* = 8.4), Earthquake Research Institute, University of Tokyo, EIC note in http://wwweic.eri.u-tokyo.ac.jp/EIC/EIC_News/index.html; 2003 Tokachi-Oki, Japan, (*M_W_* = 8.3), Yamanaka and Kikuchi (2003); 1994 Alaska (*M_W_* = 9.2), Kikuchi and Fukao (1987) and Kikuchi and Ishida (1993).

First we investigate one-dimensional (1-D) faults. For illustration purposes, Fig. 9a shows *Ṁ*_0_(*t*) for 3 earthquakes. Since the very beginning of *Ṁ*_0_(*t*) is not always determined very well, the origin of the time axis is taken at the time when *Ṁ*_0_(*t*) ≥ 5 × 10^17^ N-m for all *Ṁ*_0_(*t*) shown here. The 1994 Northridge, California earthquake (*M_W_* = 6.7) is a moderate-size earthquake having a triangular moment-rate function with a total duration of 7 sec. It is not a 1-D rupture, but we use it for illustration purposes. The triangular shape indicates that the rupture spreads out gradually from the hypocenter and stopped gradually. The 2001 Kunlun, China earthquake (*M_W_* = 7.6) is a much larger earthquake. The moment rate is relatively low, comparable to that of the Northridge earthquake, for the first 40 sec, but rapidly increases at about 45 sec. In contrast, the moment rate of the 1992 Nicaragua earthquake stays more or less constant below 5 × 10^18^ N-m/sec during the entire rupture.

The change in *Ṁ*_0_ can be due to many factors. For a 1-D fault, we write *S* = *wl*, where *l* and *w* are the fault length and width, respectively. We assume that *w* is constant for a 1-D fault. Then, the moment rate is *Ṁ*_0_ = *μ Dwl̇*= *μ DwV*. In interpreting the actual data in a simple 1-D model, *D* is taken to be the average value over the depth. In this case, an increase in *Ṁ*_0_ reflects an increase in *D* or *V* during rupture propagation. The sudden increase in *Ṁ*_0_(*t*) for the Kunlun earthquake at about 45 sec is due to increase in *D* or *V*, or both. For the Nicaragua earthquake, the relatively constant *Ṁ*_0_(*t*) suggests a smooth rupture.

Figure 9b includes *Ṁ*_0_(*t*) for 5 more recent, large earthquakes. The 1999 Chi-Chi, Taiwan earthquake, the 1999 Izmit, Turkey earthquake and the 1999 Hector Mine, California earthquake are bilateral faulting and *Ṁ*_0_(*t*) includes contributions from the two segments. The temporal variations of *Ṁ*_0_(*t*) are very complex and no obvious systematic pattern can be found. The variability becomes even more spectacular when *Ṁ*_0_(*t*) for the 1998 Balleny Is., Antarctica earthquake is added (Fig. 9c). The Balleny Is. earthquake is an unusual intra-plate earthquake which occurred in a normally aseismic, probably high stress, area (Kreemer and Holt, 2000; Conder and Forsyth, 2000; Tsuboi *et al*., 2000; Henry *et al*., 2000). The moment rate increases very rapidly and reaches a large value, 7 × 10^19^ N-m/sec.

The shape of *Ṁ*_0_(*t*) for large thrust earthquakes is expected to be different because the rupture spreads more or less two dimensionally for these events. The fault model, like the one proposed by Sato and Hirasawa (1973), yields *Ṁ*_0_(*t*) ∝*V*^3^*t*^2^ (quadratic in time). Figure 9d and 9e show several examples. Note that *Ṁ*._0_(*t*) for these earthquakes grows very large. For the recent Tokachi-Oki, Japan earthquake it reaches 6 × 10^19^ N-m/sec and for the 1964 Alaskan earthquake, it reaches 9 × 10^20^ N-m/sec.

Despite the overall large variability of *Ṁ*._0_(*t*) shown in Fig. 9a to 9e, the variation of the average slope of *Ṁ*_0_(*t*) during the first 3 sec is relatively small, within a factor of 2 to 3 as shown in Fig. 9f. Most large crustal earthquakes seem to begin more or less in the same way, but after 3 to 5 sec, the behavior becomes chaotic.

**Fig. 10:**
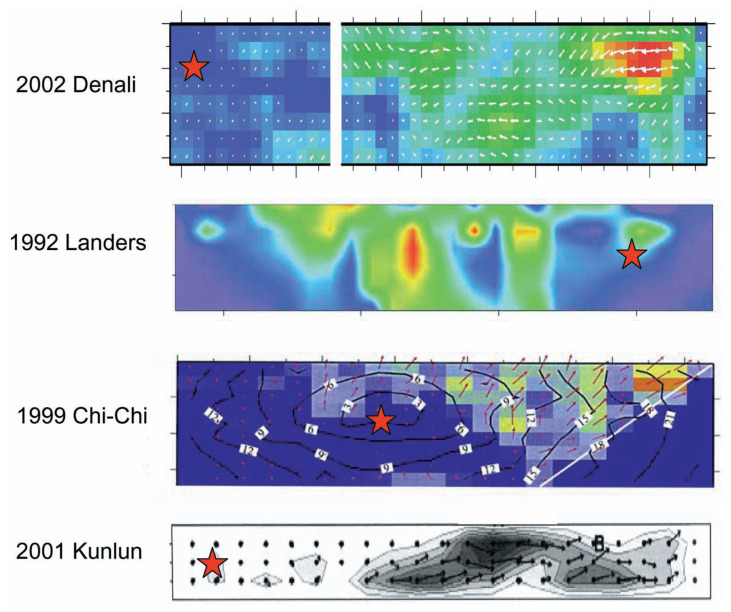
Slip distributions of recent large earthquakes. The hypocenter is indicated by a red star. Red colored areas (Denali, Landers, and Chi-Chi) and dark area (Kunlun) indicate the areas of large slip. References: Denali (Tsuboi *et al*., 2003), Landers (Wald and Heaton, 1994), Chi-Chi (Ji *et al*., 2003), Kunlun (Lin *et al*., 2003).

A more detailed inspection of the 2-D spatial rupture patterns of these earthquakes reveals that for many earthquakes some changes in the focal mechanism are involved in the beginning and the rupture near the hypocenter is relatively small compared with that at some distance away from the hypocenter (e.g., the 1992 Landers (Wald and Heaton, 1994), the 2001 Kunlun (Lin *et al*. (2003), Antolik *et al*. (2004)), the 2002 Denali (Tsuboi *et al*. (2003), Hreinsdóttir *et al*. (2003), Dreger *et al*. (2004)), and the 1999 Chi-Chi earthquakes (Ji *et al*. (2003))). Several examples are shown in Fig. 10. These cases suggest that the rupture started as a relatively small earthquake and is dynamically driven to a much larger event.

This result suggests that the earthquake rupture process is essentially a highly chaotic process and it would be difficult to predict the overall behavior from the beginning of rupture. For most shallow earthquakes with high *η_R_*, the critical fracture energy is so low that the rupture propagation is affected significantly by even minor perturbations in the strength and stress along the fault and by the dynamic effects caused by rupture propagation, resulting in highly chaotic behavior.

In contrast, the smooth *Ṁ*_0_(*t*) for the 1992 Nicaragua earthquake, an event with very low *η_R_*, suggests that the large energy dissipation associated with the fault extension acts to suppress the chaotic behavior. In the following, we elaborate on this point using a simple fracture mechanics model.

### Interpretation in terms of fracture mechanics

It is not possible to interpret the complex rupture patterns of earthquakes with a simple fracture mechanics model. Nevertheless, several basic properties of fracture mechanics provide clues to the complexity of earthquake rupture patterns.

For a simple crack, the crack extension is controlled by two quantities: the energy release rate, or crack extension force, *G*, and the critical fracture energy of the medium near the crack tip, *G_C_*. For illustration purposes, we consider a 2-D Mode III crack with length 2*c* extending at speed *V* under uniform stress *σ*_0_ with residual friction *σ_f_* on the crack surface. Then *G* is given by (Kostrov, 1966; Rose, 1976),

[17] $$G=G^{*}g(V)$$

where G* is the static energy release rate and *g*(*V*) is given by,

[18] $$g(V)={\left[(1-V/\beta )/(1+V/\beta )\right]}^{1/2}$$

where *β* is the shear-wave speed. *g*(*V*) = 0 and 1 for *V* = *β* and *V* = 0, respectively. The static energy release rate G* is approximately given by (Rose, 1976),

[19] $$G^{*}=\pi c{\left(\sigma_{0}-\sigma_{f}\right)}^{2}/2\mu$$

Whether a crack extends or stops is controlled by the balance between *G* and *G_C_*. The behavior of a crack propagating at speed *V* is governed by the equation of motion

[20] $$G=G_{C}$$

**Fig. 11:**
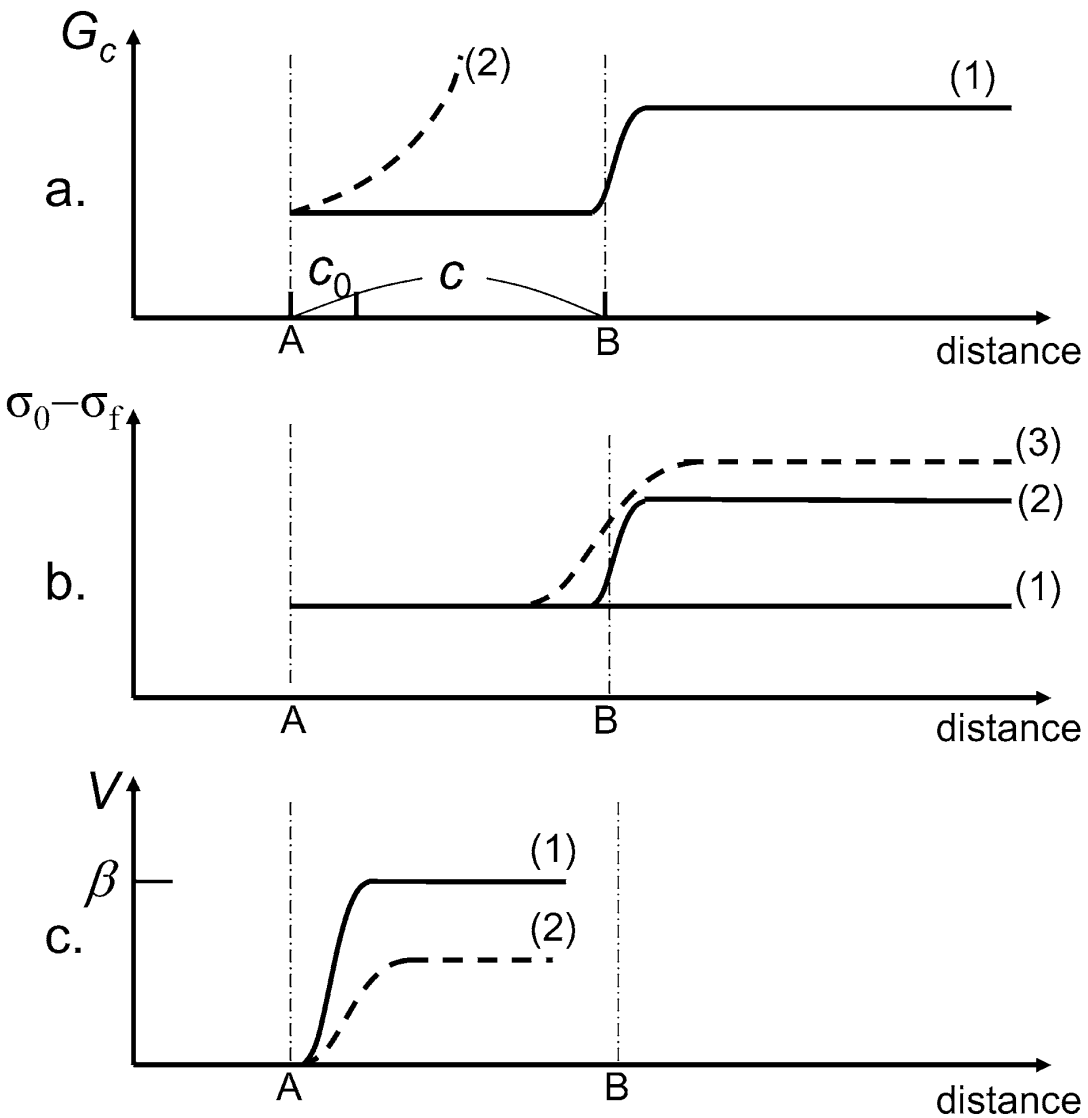
A schematic figure showing the distribution of (a) the critical fracture energy, (b) the effective driving stress, and (c) the rupture speed along a fault. An earthquake is nucleated at point A and propagates to point B.

We apply this basic physical model to understand the complex earthquake rupture patterns as shown in Fig. 11.

We assume that an earthquake nucleates at point A, with a nucleation half-length *c*_0_, and the critical fracture energy, *G_C_*, varies along the fault as shown by curve (1) in Fig. 11a. This simulates the situation in which the fault strength abruptly increases at point B. Figure 11b shows the distribution of the effective driving stress, (*σ*_0_ – *σ_f_*). Curve (1) is for a uniform distribution. Curve (2) is for a heterogeneous case in which (*σ*_0_ – *σ_f_*) increases corresponding to *G_C_*. It is reasonable to assume that (*σ*_0_ – *σ_f_*) is higher along the fault with increased strength.

The Griffith failure criterion is given by G* = *G_C_* from which

[21] $$G_{C}=\frac{\pi c}{2\mu }{\left(\sigma_{0}-\sigma_{f}\right)}^{2}$$

If this condition is satisfied at point A with *c* = *c*_0_, then the fault begins to grow. If *G_C_* is constant, as shown by curve (1) in Fig. 11a, *V* quickly approaches the limiting speed *β*, as shown by curve (1) in Fig. 11c. As the rupture reaches point B, it encounters an obstacle (i.e., barrier) represented by the sudden increase in *G_C_*. If G* < *G_C_* at point B, the rupture will stop there. ( If *G* < *G_C_*, then the rupture may keep going at a reduced speed.) This case corresponds to the Northridge earthquake shown in Fig. 9a. In contrast, if G* > *G_C_* at point B, then the fault keeps growing beyond point B. If (*σ*_0_ – *σ_f_*) increases at point B, as shown by curve (2) in Fig. 11b, the slip, *D*, will increase and the moment-rate, *Ṁ*_0_(*t*), will increase. This case can represent the behavior of most large earthquakes shown in Fig. 9b and 9c. If the friction drops as the slip increases, as discussed earlier, (*σ*_0_ – *σ_f_*) may increase more rapidly as shown by curve (3) in Fig. 11b and the variation of *Ṁ*_0_(*t*) becomes more drastic.

If *G_C_* increases with *V* during nucleation, as shown by curve (2) in Fig. 11a, then equation of motion (equation [20]) gives a rupture speed *V*, which is significantly lower than *β*, as shown by curve (2) in Fig. 11c. This corresponds to the case of an earthquake with low *V* and *η_R_*, like the 1992 Nicaragua earthquake shown in Fig. 9a.

The interpretation presented here is inevitably qualitative, but it is based on the general theory of dynamic cracks and provides a useful framework for understanding the overall behavior of earthquake rupture.

### Implications

For many earthquakes, the slip near the hypocenter is relatively small and large slip occurs sometime later at locations far from the hypocenter (Fig. 10). As discussed above, this feature may suggest that an earthquake nucleates at a weak spot and may grow if the rupture dynamics is favorable. If nucleation is caused by local weakening of the crust, due to some effects like fluid migration into the impending nucleation zone, some premonitory phenomena may be observed. Detection of such premonitory phenomena is of course important for any prediction purposes, but because of the chaotic nature of rupture discussed above, it would be probably difficult to relate it in a deterministic way to the occurrence and growth of an earthquake. Nevertheless, a better understanding of these processes is an important scientific endeavor.

## 8. Implications for earthquake damage mitigation

Seismology has an important role in reducing the impact of earthquakes on our society. Accurate predictions of earthquakes, if possible, would be obviously effective for reducing the loss of human lives caused by earthquakes. Unfortunately, as we have seen in the preceding sections, the nucleation and rupture processes of earthquakes are complex and chaotic so that it is difficult to make accurate predictions of earthquakes even if the physics of earthquakes is well understood. Another practical way to use seismology for effective damage mitigation is in earthquake “early warning”; whereby, after the occurrence of an earthquake, we rapidly estimate the severity of seismic shaking and send the information to the users at some distance away before damaging strong ground shaking begins there. Recent reviews of this subject are in Lee and Espinosa-Aranda (2002) and Kanamori (2004).

Whether we can estimate the eventual size or the characteristics of an earthquake from the very beginning of the rupture process is a basic scientific question. Seismic fault motion generates both *P*- and *S*- waves, but *P*-wave amplitude is, on average, much smaller than *S*-wave amplitude. For a point double-couple source the ratio of the maximum *P*-wave amplitude to that of the *S* wave is approximately 0.2. Thus, the *P*-wave seldom causes damage and the *S*-wave is primarily responsible for earthquake shaking damage. However, the wave form of *P*-wave reflects how the slip on the fault plane is occurring. In other words, the *P*-wave carries information and the *S*-wave carries energy. If we observe the beginning of the *P*-wave even for a short time after the onset, we can have the information on the source at least during this time period. In fact, this concept has long been used by Nakamura (1988) in the UrEDAS system for the Japanese railways.

At first glance of Fig. 9a to 9e, it appears difficult to estimate the overall size of an earthquake from the very beginning (e.g., the first 3 sec from the onset). However, Fig. 9f shows the first 15 sec of *Ṁ*_0_(*t*) and suggests that we may get some information on the total size of an earthquake, even from the first 3 sec. For example, *Ṁ*_0_(*t*) for the 1994 Northridge earthquake (*M_W_* = 6.7) stops growing at about 3 sec. In contrast, *Ṁ*_0_(*t*)’s for events larger than the Northridge earthquake are still growing at 3 sec, and it is possible to tell that the event will probably become larger than the Northridge earthquake, i.e., larger than *M_W_* = 6.7. Beyond this point, it is not obvious how large the event is going to grow. Nevertheless, despite the complexity of the moment-rate functions shown in Fig. 9a to 9e, it appears possible to estimate the lower bound of an earthquake from the first 3 sec.

**Fig. 12:**
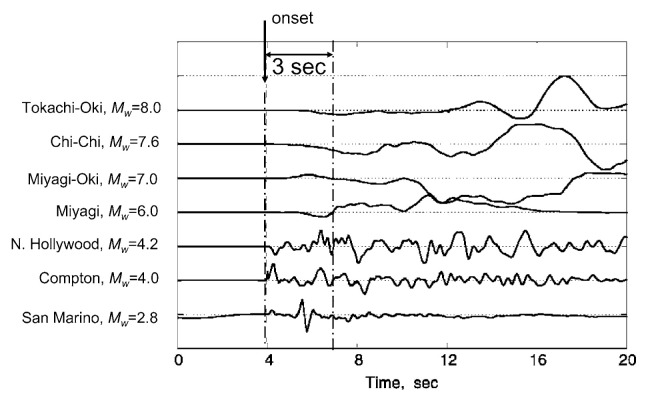
The wave forms of the beginning of close-in records of earthquakes with magnitudes from 2.8 to 8. The amplitudes are on arbitrary scales. The first 3 sec is indicated by two dash-dot lines.

Figure 12 shows close-in (i.e., short distance) records from earthquakes with magnitudes from *M* = 2.5 to 8.0. All the displacement records are filtered with a high-pass causal Butterworth filter, with a cut-off frequency of 0.075 Hz. The first 3 sec from the onset of the *P*-wave is indicated by two vertical dash-dot lines. The wave forms of large events are distinct from those of small earthquakes, suggesting that even from the first 3 sec we can make some estimation of the magnitude of the event.

**Fig. 13:**
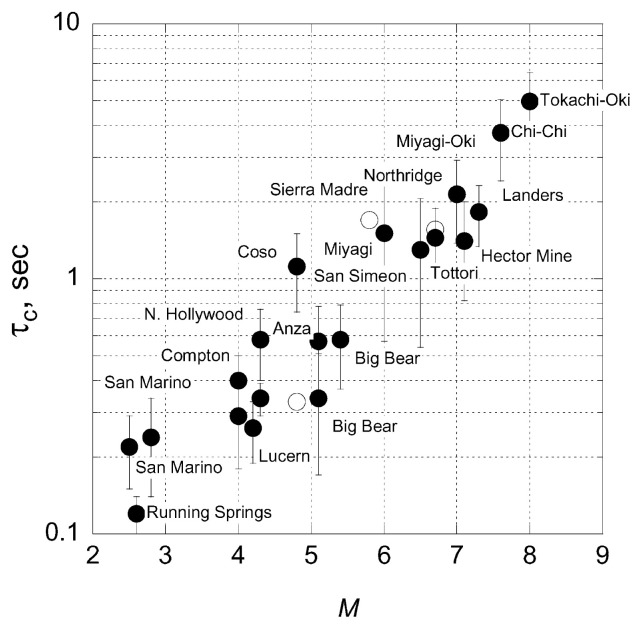
*τ_C_* computed for earthquakes with 2.5 < *M* < 8.0 in California, Japan, and Taiwan using close-in seismograms. *M* represents *M_W_* and *M_L_* (local magnitude) for *M* ≥ 6 and *M* < 6, respectively.

In general, the larger the event, the longer the period of the beginning. As a measure of the period we use a parameter, *τ_C_*, which is similar to the one used by Nakamura (1988). This parameter is determined as follows: First we compute *r* by,

[22] $$r=\frac{\int_{0}^{\tau_{0}}{\dot{u}}^{2}(t)\;dt}{\int_{0}^{\tau_{0}}u^{2}(t)\;dt}$$

where *u*(*t*) is the ground-motion displacement and the integration is taken over the time interval (0, *τ*_0_) after the onset of the *P*-wave. Usually, *τ*_0_ is set at 3 sec. Using Parseval’s theorem,

[23] $$r=\frac{4\pi^{2}\int_{0}^{\infty }f^{2}{|\hat{u}(f)|}^{2}df}{\int_{0}^{\infty }{|\hat{u}(f)|}^{2}df}=4\pi^{2}\langle f^{2}\rangle$$

where *f* is the frequency, *û*(*f*) is the frequency spectrum of *u*(*t*), and < *f* ^2^> is the average of *f* ^2^ weighted by |*û*(*f*)|^2^. Then,

[24] $$\tau_{C}=\frac{1}{\sqrt{\langle f^{2}\rangle }}=\frac{2\pi }{\sqrt{r}}$$

can be used as a parameter which represents the “period” of the initial portion of the *P* wave. Figure 13 shows *τ_C_* computed for the available close-in seismograms (Kanamori, 2004). Somewhat surprisingly, *τ_C_* keeps increasing even for earthquakes with *M_W_* > 7, without any obvious sign of saturation. Since the data set is sparse for very large events (only 5 earthquakes with *M_W_* ≥ 7), this result is not conclusive. Either the trend for *M_W_* ≥ 7 is fortuitous or the waveforms of larger earthquakes contain more long-period energy than that of smaller earthquakes. As shown by Fig. 9a, 9c, and 9d, for the Kunlun, the Balleny Is., and the Peru earthquakes, the moment rate became very large during rupture. Consequently, this method may not yield a correct magnitude. We cannot determine *τ_C_* for these earthquakes because no close-in records are available. More close-in data are obviously needed to further investigate this problem, but Fig. 13 suggests that *τ_C_* measured from only the first 3 sec of *P*-wave can be used to estimate at least the lower bound of the magnitude. In short, if *τ_C_* < 1 sec, the event has already ended or is not likely to grow beyond *M_W_* > 6. In contrast, if *τ_C_* > 1 sec, it is likely to grow beyond *M_W_* = 6. If *τ_C_* > 3 sec, the event is probably larger than *M_W_* = 7, but how large it will eventually become cannot be determined.

At present, the technology of earthquake early warning is still in progress, but the best way to assess the robustness and utility of the early warning concept is to implement it on an existing seismic network for real-time testing. Large earthquakes are relatively rare and it is important to gain experience with more frequent, smaller earthquakes. As more new methods are implemented and tested real-time, we will discover a novel usage of reliable earthquake early warning information which will significantly contribute to effective earthquake damage mitigation.

## 9. Conclusion

The energy-based, rather than empirical, quantification of earthquakes in terms of *M_W_* clarified some important features of spatial (Fig. 1) and temporal (Fig. 2) variations of global seismicity which were not seen with the old quantification methods.

The ratio, *η_R_*, of the radiated energy to the sum of radiated and fracture energies during a seismic rupture controls the characteristics of an earthquake. Some earthquakes, like slow tsunami earthquakes and large deep earthquakes, involve highly dissipative processes during faulting and have small *η_R_*. In contrast, most shallow, large earthquakes appear to have large *η_R_* and, in this sense, they are “brittle” events. Here, the term “brittle” is used to mean that the resistance to rupture is weak and a rupture, once started, can grow rapidly and is hard to stop. Because of the weak resistance, even minor heterogeneities in the strength and stress along a fault can perturb the rupture propagation and cause a chaotic behavior of fault ruptures, seen in the moment rate functions of many large, shallow earthquakes.

It is difficult to predict the nucleation and growth process of earthquakes because of the chaotic behavior. This leads to the difficulty in making accurate predictions of earthquakes, even if the basic physics of earthquakes is understood well. Given this difficulty, an effective approach to earthquake damage mitigation is through “early warning” in which the eventual size of an earthquake is estimated from the very beginning of the *P*-wave so that an early warning of the damaging ground motion due to the *S*-wave can be issued. Because a seismic faulting is essentially a shear faulting, the first arriving *P*-wave is small, and seldom causes damage. The late arriving *S*-wave carries energy and causes damage. Taking advantage of this special property of energy radiation from a seismic faulting, we can develop effective early warning methods which will play an important role in modern societies with large and sophisticated structures.

The quality of the determinations of earthquake parameters is improving rapidly with the recent advancement in seismic instrumentation and computational facility, yet, as mentioned many times in this paper, the uncertainties are still large and often significantly different results are obtained by different investigators. Inevitably some subjective judgments have to be made in choosing the results in the literature to develop the models presented in this paper. A significant improvement in seismological practice and a good understanding of the basic physics of earthquakes is central to the development of seismology in the future.
